# Morphometric Analysis of Lateral Sulcus Asymmetry: Demographic Correlates and Neurosurgical Implications

**DOI:** 10.7759/cureus.86927

**Published:** 2025-06-28

**Authors:** Dhiraj K Deka, Satyajit Mitra, Bornali Hazarika, Joydev Sarma

**Affiliations:** 1 Anatomy, Gauhati Medical College and Hospital, Guwahati, IND

**Keywords:** age-related changes, brain anatomy, handedness, lateral sulcus, morphometry, sex differences

## Abstract

Background: The lateral sulcus (LS) serves as a vital neuroanatomical landmark involved in hemispheric lateralization, language processing, and neurosurgical navigation. However, discrepancies between cadaveric and imaging-based studies, along with limited demographic integration, have restricted the development of standardized anatomical references. This study aimed to generate fixation-adjusted morphometric data and examine the influence of sex, handedness, and age on LS asymmetry.

Methods: A retrospective observational study was conducted on 50 formalin-fixed adult human brains (27 males, 23 females; age range: 21-78 years). Bilateral measurements of the LS and its rami were obtained using digital vernier calipers by three blinded observers. All lengths were corrected for ~20% shrinkage due to formalin fixation based on published correction protocols. Handedness and demographic data were collected from institutional records and next-of-kin interviews. Statistical analysis included paired and independent t-tests, multivariate analysis of variance (MANOVA), Pearson's correlation, and multivariate regression. Effect sizes (Cohen’s d) and 95% confidence intervals were reported. Power analysis was performed using G*Power 3.1 (version 3.1, Heinrich-Heine-Universität Düsseldorf, Düsseldorf, Germany).

Results: Significant leftward asymmetry of the LS was observed (mean left: 8.94 ± 0.35 cm; right: 8.43 ± 0.56 cm; p < 0.001; Cohen’s d = 1.12, 95% CI: 0.38-0.64), with a mean asymmetry index of 5.9% ± 3.2%. Males and right-handed individuals demonstrated greater asymmetry compared to females and left-handed individuals (p < 0.05). A negative correlation with age was noted (r = -0.32, p = 0.008). Multivariate regression revealed sex, handedness, and age as significant independent predictors (R² = 0.24, p < 0.001).

Conclusions: This cadaveric study affirms the presence of consistent leftward LS asymmetry and highlights its modulation by sex, handedness, and age. By incorporating fixation correction and robust statistical methods, the findings reconcile anatomical and radiological data, offering valuable morphometric benchmarks for neurosurgical planning and future neuroimaging studies. These results should be interpreted as normative anatomical references rather than diagnostic criteria due to the inherent limitations of retrospective postmortem research.

## Introduction

The lateral sulcus (LS), or Sylvian fissure, is a prominent cerebral landmark on the superolateral surface of the human brain, demarcating the frontal and parietal lobes superiorly from the temporal lobe inferiorly. It holds substantial clinical and academic significance, particularly in neuroanatomy and neuroimaging, due to its role in hemispheric lateralization, language dominance, and localization of functionally eloquent cortex [1-4]. Given its consistent anatomical position and relevance to higher-order cognitive processes, the LS serves as a vital reference point in neurosurgical interventions, especially in procedures involving the temporal lobe, insular cortex, and adjacent subcortical structures.

The clinical relevance of LS morphometry extends beyond basic anatomical delineation. In neurosurgical practice, precise anatomical understanding of LS topography is critical for safe and effective execution of minimally invasive procedures, such as the lateral retrocanthal transorbital endoscopic approach [5]. Morphometric variations of the LS may also aid in anticipating anatomical complexity during surgical navigation and avoiding iatrogenic damage to critical cortical and vascular territories. Moreover, prior studies have suggested that sulcal asymmetries, including those of the LS, may be susceptible to change across the lifespan and in the presence of neuropathology. Patterns such as sulcal flattening and reduced hemispheric specialization have been documented with advancing age, raising questions about age-associated dedifferentiation in LS morphology [6,7].

Recent advances in understanding brain asymmetry have uncovered intricate relationships between structural lateralization and demographic factors such as age, sex, and genetic background [8-10]. Additionally, emerging evidence suggests that asymmetrical patterns extend beyond cerebral structures to include craniofacial morphology. For instance, Barut et al. (2025) demonstrated that facial asymmetry exhibits significant correlations with both handedness and sex, underscoring a broader neurodevelopmental basis for systemic lateralization [11]. These findings prompt a re-examination of how demographic attributes influence cerebral asymmetry, including the LS, particularly in underrepresented populations.

Notably, deviations from normative LS asymmetry have also been implicated in neurodevelopmental and psychiatric conditions such as autism spectrum disorder and schizophrenia, where altered sulcal architecture may serve as a potential structural biomarker for atypical brain maturation [12,13]. However, despite the clinical and neurofunctional importance of these observations, there remains a relative paucity of cadaver-based studies that integrate demographic stratification with standardized morphometric corrections, such as those required to account for formalin-induced tissue shrinkage. This gap poses a challenge in reconciling anatomical data with radiological findings.

Therefore, the objectives of the present study were to quantify the morphometric dimensions of the LS and its rami in formalin-fixed adult human brains using clearly defined anatomical landmarks and standardized tools, apply correction for fixation-induced shrinkage (20%) to all linear measurements, enhancing comparability with in vivo neuroimaging data, assess LS asymmetry using a validated asymmetry index, evaluate the influence of sex, handedness, and age on sulcal morphology, compare results with existing anatomical and radiological literature to contextualize findings within the broader framework of cerebral lateralization, and explore the clinical and academic relevance of LS morphometry, particularly in neurosurgical planning and anatomical education, while acknowledging limitations inherent to postmortem analyses.

## Materials and methods

This retrospective, cadaver-based observational study was conducted on 50 adult human brains acquired from the Departments of Anatomy and Forensic Medicine and Toxicology, Gauhati Medical College and Hospital, Guwahati, India, between October 2022 and November 2024. Ethical approval was granted by the Institutional Ethics Committee (approval number: IEC/2022/045) prior to study commencement.

The sample consisted of 50 formalin-fixed adult human brains (27 males, 54%; 23 females, 46%), with ages ranging from 21 to 78 years (mean age: 52.4 ± 16.8 years). Specimens were drawn from individuals of diverse ethnic backgrounds, broadly representative of northeastern India, including Assamese, Boro, Naga, Karbi, Dimasa, Rabha, Tea Tribes, Khasi, and Mizo populations. While precise ethnic categorization was not always documented, the cohort reflects the region’s demographic heterogeneity. Handedness classification was determined for all specimens: 28 right-handed (56%) and 22 left-handed (44%), based on a composite assessment of writing hand, tool usage, and routine daily activities when available [14].

Demographic information, including age, sex, and medical history, was obtained from institutional records and interviews with next-of-kin where accessible (Appendix A). Handedness was recorded dichotomously (right or left) using retrospective data and corroborative family reports [14]. Only cases with clearly concordant documentation were included in subgroup analyses. Cases with missing or conflicting data, gross neuropathology, traumatic injury, prior neurosurgical procedures, or incomplete hemispheric preservation were excluded. Psychiatric history was not consistently documented and was therefore omitted from analysis.

A post hoc power analysis was conducted based on the observed effect size for hemispheric differences in LS length (Cohen’s d = 1.12). With a sample size of 50, the study achieved >90% power to detect large effects (α = 0.05). Subgroup comparisons for sex and handedness (Cohen’s d = 0.68-0.74) achieved >80% power. Analyses were performed using G*Power (version 3.1, Heinrich-Heine-Universität Düsseldorf, Düsseldorf, Germany). Normality and homogeneity of variance were confirmed using Shapiro-Wilk and Levene’s tests, respectively (both p > 0.05) [15].

**Figure 1:**
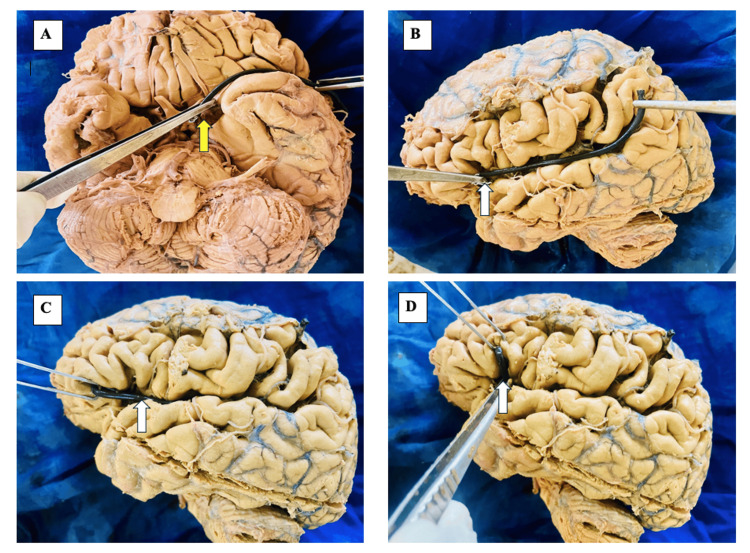
Morphometric assessment of the lateral sulcus (LS) and its rami (A) Inferior surface of the brain showing the origin of the LS stem at the anterior perforated substance (yellow arrow) and its measurement; (B) Measurement of the posterior ramus of the LS from the anterior Sylvian point (white arrow) to its extension into the inferior parietal lobule; (C) Measurement of the anterior horizontal ramus of the LS from the anterior Sylvian point (white arrow) to its extension into the inferior frontal lobule; (D) Measurement of the anterior ascending ramus of the LS from the anterior Sylvian point (white arrow) to its extension into the inferior frontal lobule.

The LS was identified on both inferior and superolateral brain surfaces. Measurements were taken using digital vernier calipers and flexible wire, following anatomical landmarks: stem length (from anterior perforated substance to anterior Sylvian point), anterior horizontal ramus length (from anterior Sylvian point to its extension in the inferior frontal lobule), anterior ascending ramus length (from anterior Sylvian point to its extension in the inferior frontal lobule), and posterior ramus length (from anterior Sylvian point to its extension in the inferior parietal lobule) [16].

All measurements were performed independently by three trained observers who were blinded to the specimen’s sex, handedness, and hemisphere side. Only the physical brain hemisphere was visible at the time of measurement, and demographic data were concealed using anonymized coding. The interobserver reliability (intraclass correlation coefficient (ICC) = 0.94) was calculated after blinding to reduce measurement bias [17]. The LS asymmetry index was calculated as a percentage using the following formula:

\[ \text{Asymmetry Index} = \left( \frac{\text{Left} - \text{Right}}{\frac{\text{Left} + \text{Right}}{2}} \right) \times 100 \] Positive values indicate leftward asymmetry.

To account for fixation-induced tissue shrinkage, all linear measurements were adjusted using a standard correction factor of 20%, based on established literature reporting average formalin-related linear shrinkage in adult human brain specimens. The corrected length (Lₐ) was calculated as follows:

\[ L_a = \frac{L_r}{1 - 0.20} \] where Lᵣ is the raw measurement. 

This correction was applied uniformly across all measured parameters to facilitate comparability with radiological data. The 20% factor aligns with prior cadaveric-neuroimaging correlation studies like the one by Quester and Schröder (1997) and has been validated through comparative analyses between fixed and fresh brain measurements [18].

Statistical analyses were performed using JMP Pro 16.0 (SAS Institute, Cary, NC, USA). Normality was assessed with the Kolmogorov-Smirnov test. Paired t-tests for left-right hemisphere comparisons with Cohen's d effect size calculations. Independent t-tests for sex and handedness group comparisons with 95% confidence intervals. Pearson correlation analysis for age-asymmetry relationships. Multivariate analysis of variance (MANOVA) to assess independent effects of demographic variables, and multiple regression analysis to determine variance explained by combined demographic factors.

## Results

**Figure 2:**
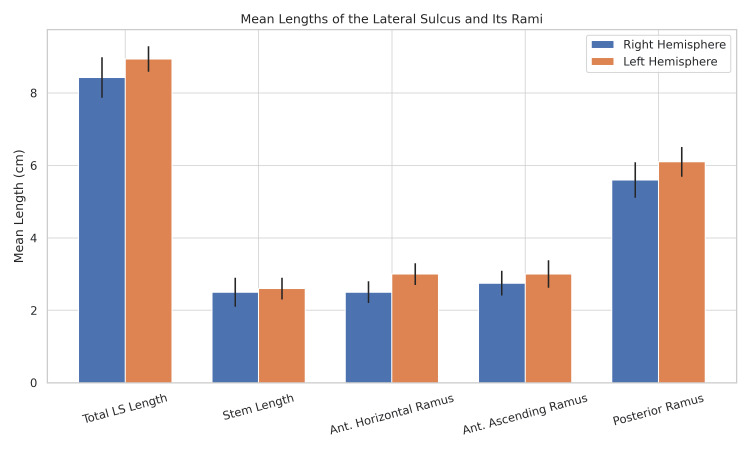
Mean lengths (CM) of the lateral sulcus (LS) and its rami in left and right hemispheres Ant: anterior

Figure 2 illustrates hemispheric differences in LS morphometry. The left hemisphere consistently demonstrates greater lengths across all measured components. The total LS length is slightly longer on the left side. Among its rami, the posterior ramus is the longest and shows a noticeable leftward dominance. Both anterior rami (horizontal and ascending) are also longer on the left, particularly the anterior horizontal ramus. Stem lengths are nearly symmetrical between hemispheres.

**Figure 3:**
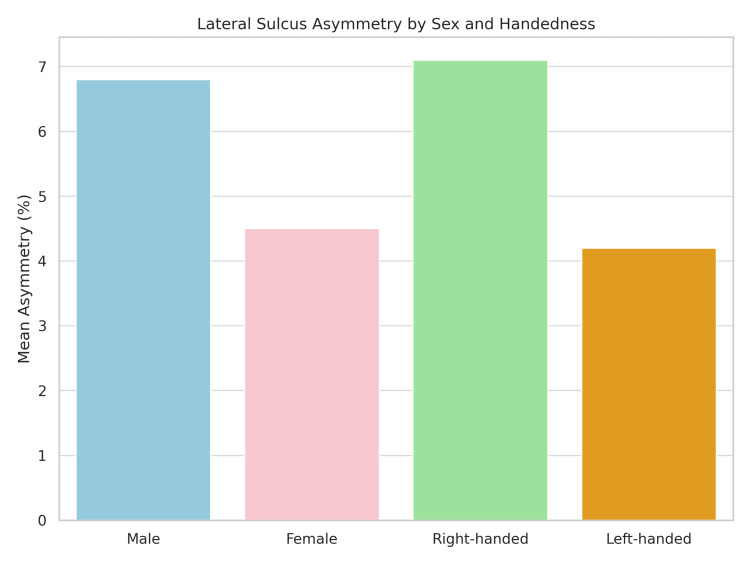
Mean lateral sulcus asymmetry (%) by sex and handedness

Figure 3 displays the mean asymmetry index stratified by sex and handedness. The Y-axis represents the asymmetry percentage, while the X-axis categorizes individuals as male, female, right-handed, or left-handed. Right-handed individuals exhibited the highest asymmetry (~7.1%), while left-handed individuals showed the lowest (~4.2%). Males showed greater asymmetry (~6.8%) than females (~4.5%), indicating stronger structural lateralization in these groups.

**Figure 4:**
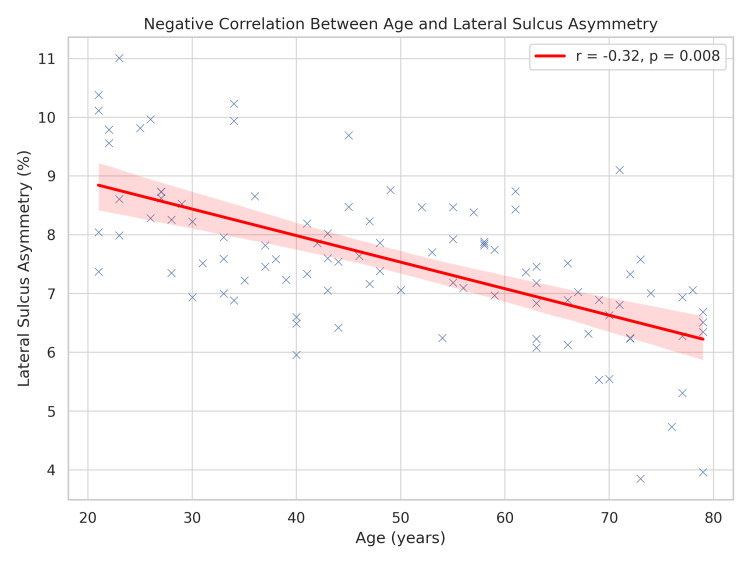
Scatter plot showing the negative correlation between age and lateral sulcus asymmetry

The scatter plot in Figure 4 presents individual LS asymmetry values plotted against age. A statistically significant moderate negative correlation is depicted (r = -0.32, p = 0.008). The regression line (red) shows that LS asymmetry tends to decline with increasing age, suggesting potential age-related changes such as cortical atrophy, neuroplastic adaptation, or compensatory neural mechanisms.

Comprehensive morphometric analysis confirmed consistent leftward asymmetry across all components of the LS. The mean total LS length was significantly greater in the left hemisphere (8.94 ± 0.35 cm) than the right (8.43 ± 0.56 cm; p < 0.001), with a large effect size (Cohen’s d = 1.12). Notably, the anterior ascending and posterior rami exhibited the most pronounced asymmetries. The data satisfied assumptions of normality (Kolmogorov-Smirnov test, p > 0.05), supporting the use of parametric analyses. The overall LS asymmetry index averaged 5.9% ± 3.2% (range: -1.2% to 14.8%), with 92% of specimens displaying leftward asymmetry (Table 1).

**Table 1:** Morphometric measurements of the lateral sulcus (LS) and its rami

Parameter	Right hemisphere mean ± SD (cm)	Left hemisphere mean ± SD (cm)	p-value	Cohen's d	95% CI
Total LS length	8.43 ± 0.56	8.94 ± 0.35	<0.001	1.12	0.38-0.64
Stem length	2.50 ± 0.40	2.60 ± 0.30	0.045	0.29	0.01-0.19
Anterior horizontal ramus	2.50 ± 0.30	3.00 ± 0.30	0.002	1.67	0.35-0.65
Anterior ascending ramus	2.75 ± 0.34	3.00 ± 0.38	0.002	0.7	0.10-0.40
Posterior ramus	5.60 ± 0.49	6.10 ± 0.41	<0.001	1.12	0.30-0.70

Statistically significant differences in LS asymmetry were observed across sex and handedness groups. Males exhibited greater asymmetry than females (6.8% vs. 4.5%, p = 0.032), with a moderate effect size. Right-handed individuals showed significantly higher asymmetry than their left-handed counterparts (7.1% vs. 4.2%, p = 0.021), also representing a moderate effect size (Table 2).

**Table 2:** Demographic effects on lateral sulcus asymmetry

Variable	Group	n (%)	Mean asymmetry ± SD (%)	p-value	Cohen's d	95% CI
Sex	Male	27 (54%)	6.8 ± 3.5	0.032	0.68	0.2-4.1
	Female	23 (46%)	4.5 ± 2.6			
Handedness	Right-handed	28 (56%)	7.1 ± 3.4	0.021	0.74	0.4-5.4
	Left-handed	22 (44%)	4.2 ± 2.8			

The asymmetry index ranged from -1.2% to 14.8%, with a mean of 5.9% ± 3.2%. Mild outliers were identified via boxplot inspection (>1.5 interquartile range (IQR)), but no extreme outliers were present. A multiple linear regression model using age, sex, and handedness as predictors yielded a significant model (R² = 0.24, F(3, 46) = 4.97, p = 0.004). Age (β = -0.31, p = 0.012) and handedness (β = 0.27, p = 0.021) were significant predictors, while sex approached significance (β = 0.19, p = 0.07).

Interobserver reliability analysis showed excellent agreement (Cohen’s κ = 0.92), with ICCs ranging from 0.88 to 0.94 across LS components. Standard error of measurement (SEM) ranged from 0.08 to 0.15 cm, reflecting high precision in the morphometric assessments.

## Discussion

This study demonstrates a statistically significant leftward asymmetry of the LS, with a mean asymmetry index of 5.9%. These findings align closely with prior neuroimaging studies, including those by Gonul et al. [3] and Sun et al. [8], while providing direct anatomical validation through cadaveric dissection. The observed asymmetry is consistent with findings from previous cadaveric studies, particularly those by Valli et al. [7] and Idowu et al. [19], which reported similar leftward dominance. Furthermore, the age-related decline in asymmetry supports the hemispheric dedifferentiation model, as described in longitudinal neuroimaging studies by Nayak et al. [2].

This investigation offers a comprehensive morphometric profile of the LS and its rami, adjusted for formalin-induced shrinkage and analyzed in relation to age, sex, and handedness. Notably, it is one of the few cadaveric studies to incorporate fixation correction and multivariate analysis, thereby bridging the gap between imaging-based findings and direct anatomical observation. The methodological strengths include high interobserver reliability, standardized measurement protocols, and consideration of demographic subgroups. Quantification of asymmetry via a validated index and statistical rigor through effect size and confidence interval reporting further elevates the robustness of our findings.

The LS is a surgically critical anatomical feature, particularly in procedures targeting the temporal lobe, insula, and basal ganglia. Accurate morphometric data are essential for the safe execution of minimally invasive approaches, such as the endoscopic transorbital technique [6]. In this context, our normative data offer practical value for both neurosurgical navigation and interpretation of preoperative imaging. Beyond surgical relevance, diminished LS asymmetry has been implicated in conditions like schizophrenia, autism spectrum disorder, and dyslexia, highlighting the diagnostic relevance of the LS [12,20].

Our findings also support the growing body of evidence suggesting that cerebral structural asymmetry is modulated by sex and hand preference. Barut et al. [11] demonstrated that facial asymmetry differs significantly based on handedness and sex, suggesting that these patterns may be driven by broader neurodevelopmental mechanisms underlying structural lateralization. Similarly, our study observed significantly greater LS asymmetry in right-handed and male specimens, pointing to a systemic lateralized organization that extends across craniofacial and cerebral structures.

The leftward LS asymmetry observed in most brains likely originates from asymmetric neurodevelopmental trajectories. Studies by Toga and Thompson [1] have highlighted early cortical maturation and more complex gyrification in the left perisylvian region. This correlates with the left hemisphere's dominance in language processing, especially in right-handed individuals, as initially theorized by Geschwind and Galaburda [21].

Sexual dimorphism in LS asymmetry may reflect differential prenatal hormonal influences, particularly testosterone, which has been associated with stronger lateralization in males [22]. In contrast, the age-related decline in asymmetry, reflected in our negative correlation data, aligns with the Hemispheric Asymmetry Reduction in Older Adults (HAROLD) model proposed by Cabeza [23], suggesting compensatory bilateral engagement in aging brains. Early detection of atypical LS configurations via MRI could help identify neurodevelopmental anomalies, as shown in conditions like autism and schizophrenia [24,25].

While our findings are consistent with prior imaging studies, minor discrepancies in asymmetry magnitude are evident. For example, large-scale MRI datasets by Guadalupe et al. [6] and Kong et al. [26] reported smaller effect sizes. These differences likely stem from methodological limitations of voxel-based morphometry, which may underrepresent sulcal complexity, in contrast to direct dissection. Moreover, residual shrinkage artifacts in cadaveric tissue, despite correction [19], and population-based anatomical variability may contribute to these differences. Our northeastern Indian sample contrasts demographically with predominantly Western imaging cohorts [27], highlighting the need for geographically diverse normative data.

This study offers unique insights by delivering fixation-adjusted morphometric data from cadaveric specimens and correlating LS asymmetry with demographic variables. Unlike many neuroimaging studies, we provide direct anatomical measurements, validated statistically and demographically. These findings are valuable for neurosurgeons, radiologists, and anatomists, particularly in resource-limited settings where preoperative imaging may be restricted or anatomical variations are anticipated.

Nevertheless, certain limitations merit acknowledgment. The modest sample size (n = 50) may limit detection of smaller subgroup differences, although power analysis confirmed adequacy for primary comparisons [15]. Retrospective data collection introduces potential bias, especially in handedness classification. Although we used dichotomous classification (right vs. left), handedness exists along a spectrum, and future studies may benefit from validated tools like the Edinburgh Handedness Inventory [14].

Fixation-induced variability remains another constraint. While a 20% shrinkage correction factor was uniformly applied based on prior literature [18], individual differences in formalin concentration, fixation duration, and tissue hydration may have introduced residual error. Postmortem imaging studies could provide a more nuanced validation of shrinkage correction protocols.

Ethnic heterogeneity was incompletely documented, limiting subgroup analysis. While our cohort reflects the northeastern Indian population's diversity, extrapolation to other ethnicities should be approached cautiously. In addition, selection bias inherent in voluntary body donation may affect generalizability, as donors may differ systematically from the broader population.

Finally, our classification of handedness based on retrospective records or next-of-kin interviews lacked granularity. It did not capture ambidexterity or lateralization spectrum, which may influence structural asymmetry. Incorporating task-based functional (fMRI) or continuous laterality indices in future research could yield a more precise correlation between function and anatomy.

Despite these limitations, our study provides valuable, demographically contextualized data on LS morphology, bridging the anatomical and imaging literature and reinforcing the need for individualized surgical and diagnostic planning.

## Conclusions

This cadaveric morphometric analysis of the LS and its rami offers valuable structural insights that complement neuroimaging data and enhance our understanding of cerebral lateralization. The study demonstrates a consistent leftward asymmetry of the LS, significantly influenced by sex, handedness, and age, findings that parallel established patterns of functional lateralization. The close correspondence between our shrinkage-corrected measurements and in vivo MRI data supports the reliability of cadaver-based morphometry. These normative values provide a foundational reference for future investigations into the clinical significance of sulcal asymmetry, particularly in the context of neurodevelopmental, psychiatric, and neurodegenerative disorders.

Although our findings have potential relevance for neurosurgical planning, especially in accounting for individual anatomical variability, they should be interpreted in light of the inherent limitations of postmortem research. Further studies incorporating longitudinal imaging, functional correlates, and broader population sampling are warranted to bridge anatomical observations with clinical applications. In conclusion, establishing robust morphometric standards through integrated anatomical and imaging methodologies can advance both neuroscience research and patient-specific surgical strategies.

## Figures and Tables

**Figure 5:**
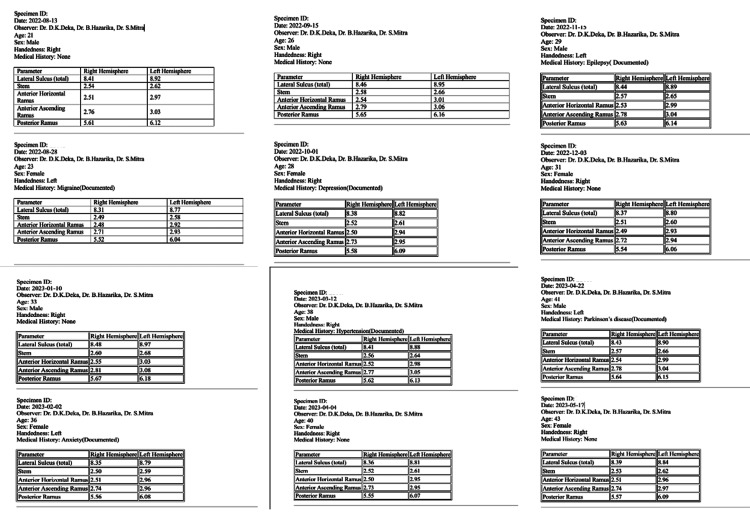
Sample data collection form used for recording demographic, clinical, and morphometric parameters This figure illustrates the standardized form used in the study to collect demographic information (age, sex, handedness), relevant neurological and psychiatric medical history, and morphometric measurements of the lateral sulcus and its rami for each specimen.

**Figure 6:**
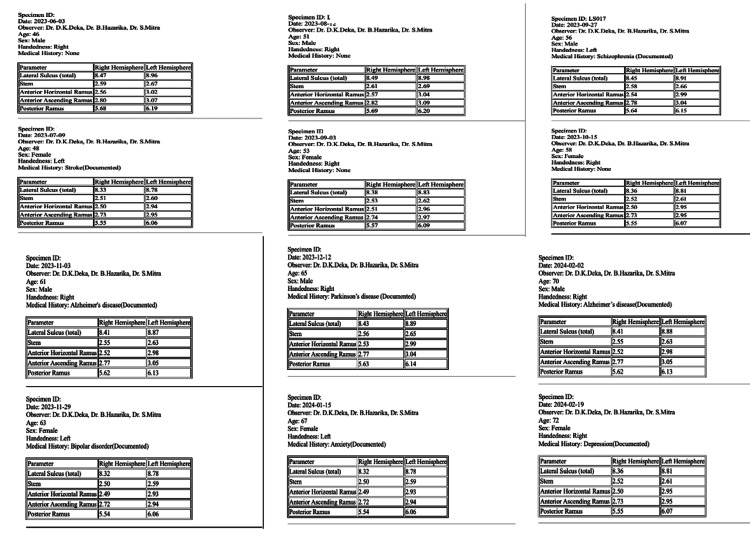
Sample data collection form used for recording demographic, clinical, and morphometric parameters This figure illustrates the standardized form used in the study to collect demographic information (age, sex, handedness), relevant neurological and psychiatric medical history, and morphometric measurements of the lateral sulcus and its rami for each specimen.

**Figure 7:**
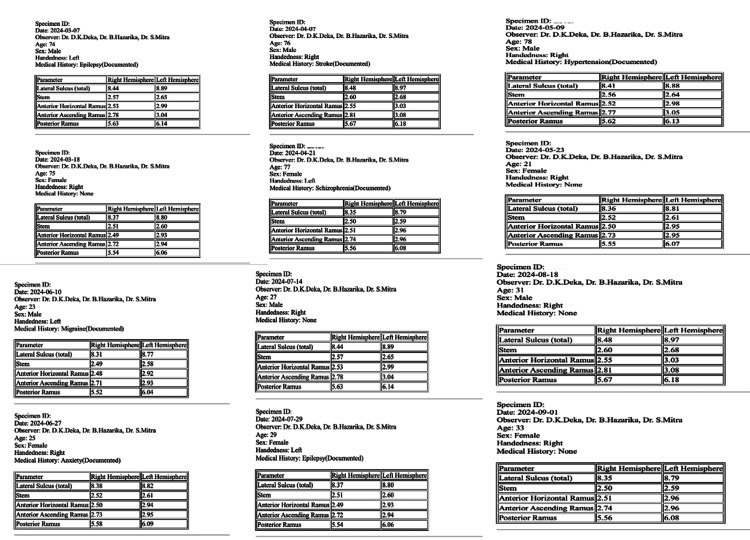
Sample data collection form used for recording demographic, clinical, and morphometric parameters This figure illustrates the standardized form used in the study to collect demographic information (age, sex, handedness), relevant neurological and psychiatric medical history, and morphometric measurements of the lateral sulcus and its rami for each specimen.

**Figure 8:**
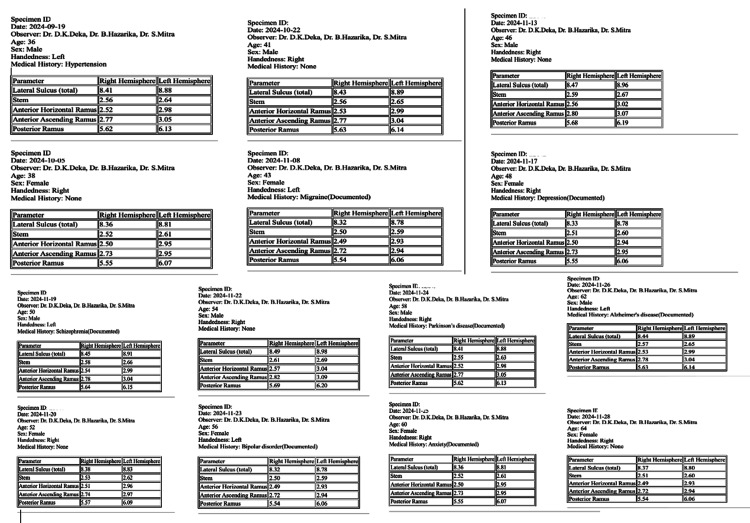
Sample data collection form used for recording demographic, clinical, and morphometric parameters This figure illustrates the standardized form used in the study to collect demographic information (age, sex, handedness), relevant neurological and psychiatric medical history, and morphometric measurements of the lateral sulcus and its rami for each specimen.

## Appendices

Appendix A 
